# Phytogenic cocktails fed in different feeding regimes as alternatives to antibiotics for improving performance, intestinal microbial, and carcass characteristics of slow growth chickens

**DOI:** 10.14202/vetworld.2024.1423-1429

**Published:** 2024-07-06

**Authors:** Tiurma Pasaribu, Arnold P. Sinurat, Marsudin Silalahi, Jonathan Anugrah Lase

**Affiliations:** Research Center for Animal Husbandry, National Research and Innovation Agency, Cibinong Science Center, Cibinong-Bogor 16915, West Java, Indonesia

**Keywords:** antibiotic, carcass, intestinal microbial, performance, phytogenic cocktail, slow growth chickens

## Abstract

**Background and Aim:**

The phytogenic cocktail (PC) is a unique combination of natural plant extracts consisting of coconut shell smoke, clove leaf extract, and mangosteen rind extract, predominantly containing phenol, eugenol, and α-mangostin. Chicken performance can be improved by its antibacterial properties. This study aimed to test PC as a replacement for antibiotic growth promoters (AGPs), assessing its impact on performance, intestinal microbes, and carcass traits in slow growth KUB chickens.

**Materials and Methods:**

Two hundred and forty KUB chicks were distributed randomly to five dietary groups. Each group constituted six replicates, one replicate contained eight chicks. The treatments included the control diet (CD) with no additives, CD with 50 ppm Zinc bacitracin as an additive (AGPs), CD paired with 198 mL PC/ton feed provided for the initial 12 weeks (PC1), CD with 198 mL PC/ton feed given for the first 4 weeks (PC2), and CD supplied with 198 mL PC/ton feed for the first 8 weeks (PC3). Performance and mortality indicators were assessed during the feeding stage up to 12 weeks of age, while intestinal total microbial count and carcass characteristics were determined at 12 weeks. Duncan’s multiple-range test identified differences among the treatments in the randomized experiment.

**Results:**

The AGPs group weighed significantly more (p < 0.05) than PC2 but not significantly different (p > 0.05) from Control, PC1, and PC3 at 4 weeks. At 8 weeks, there was no significant difference (p > 0.05) in the body weight (BW) between the AGP, CD, and PC groups. The AGPs group had a significantly greater BW than PC1 and PC2 at 12 weeks (p < 0.05), but was comparable to CD and PC3 (p > 0.05). During the starter phase (0–4 weeks), dietary addition of AGPs or PCs significantly reduced feed intake (p < 0.05); however, no significant effect (p > 0.05) was observed during the later feeding periods (0–8 or 0–12 weeks). During the starter period, PC3 yielded the best feed conversion ratio, slightly surpassing AGPs and significantly (p < 0.05) outperforming CD. No significant variations (p > 0.05) were detected in the carcasses among the treatments. The reduction of abdominal fat relative weight was significant (p < 0.05) during the first 8 weeks of PC feeding. After the 12-week trial, no significant difference (p > 0.05) was observed in the proportionate weights of the crop, proventriculus, gizzard, pancreas, cecum, spleen, bursa of Fabricius, heart, and liver. The reduction in the intestinal microbe population due to AGPs or PC was not statistically significant (p > 0.05). About 100% viability was confirmed by the absence of mortality throughout the study.

**Conclusion:**

PC supplementation in KUB chicken feed enhances their performance. The optimal feeding regimes were effective during the first 8 weeks of age. In the 0–4 week time frame, feeding the PC to the chicken worsened performance whereas no improvement was observed in the 0–12 week period. The application enhanced weight loss, feed efficiency, and reduced abdominal fat. Based on the research findings, the PC can replace AGPs as a feed additive due to comparable or superior improvement results.

## Introduction

Antibiotic growth promoters (AGPs), historically added to animal feed such as chickens, pigs, and cattle, are substances in question. AGPs have been implemented to boost productivity, enhance livestock output (meat and eggs), improve feed utilization, and fortify resistance against diseases [1]. For several decades, AGPs have been extensively used in the livestock industry. These feed additives are thought to promote animal growth through their ability to decrease harmful bacterial infections in the digestive tract, thus boosting gut microbiota and enhancing nutrient absorption. In animal agriculture, the application of AGPs has sparked controversy due to various reasons. Consuming meat from animals fed antibiotics may pose risks to human health, including allergies and antibiotic-resistant microorganisms. The emergence of antibiotic-resistant microbes in both humans and animals poses significant public health challenges due to the difficulty in treating such infections. Antibiotics excreted by animals and found in manure can lead to antibiotic-resistant microorganisms in soil and water. Due to rising concerns over antibiotics in food, there is a growing call for antibiotic-free animal products. Due to worries about AGPs, several nations have enacted rules controlling their usage in livestock farming. In several countries [2], the application of antibiotics as feed additives is restricted or prohibited. European countries banned the utilization of AGPs by 2006 [3]. Since January 2018, AGPs have been banned in Indonesia. European countries have banned the use of AGPs since 2006 [3]. Instead of using antibiotics as growth promoters, the livestock industry has turned to methods such as improved animal husbandry, superior nutrition, and phytogenic feed additives to enhance animal growth and feed efficiency. KUB (Kampung Unggul Balitbangtan) chickens, specifically bred from native chickens in West Java, Indonesia, offer superior growth and egg production due to their unique genetic advantages.

Phytogenics, sourced from herbs, spices, and other plants, serve as natural feed additives due to their distinct health-promoting properties. Phytochemicals, phytogenics, and phytobiotics are alternative names for these bioactive compounds [4]. Bioactive substances serve as antibacterial agents in most cases. Roselle, clove, rosemary, and thyme extracts exhibit antibacterial properties against *Escherichia coli* and *Staphylococcus aureus* [5]. *Avicennia marina* (Forssk.) Vierh roots’ ethanol extract can inhibit the growth of *Pseudomonas aeruginosa*, *Bacillus subtilis*, *S. aureus*, *E. coli*, *Aspergillus fumigatus*, and *Candida albicans*. Ethyl acetate extract from *A. marina* leaves can suppress growth of *S. aureus*, *E. coli*, and *B. subtilis* [6]. Excessive levels of active components such as total phenol, tannin, and saponin in the non-performing high-dose cocktail might hinder nutrient absorption [7]. A combination of coconut shell liquid smoke, clove leaf extract, and mangosteen pericarp extract has been shown to suppress *E. coli* and *Candida utilis* growth based on other *in vitro* research [8]. Bioactive substances have been reported as alternatives to AGPs in living organism research. The lowest dose of a phytogenic cocktail (PC) (cashew nut liquid smoke + *Phyllanthus niruri* extract + clove leaves extract) yielded the top performance for broilers, matching that of birds fed AGPs in a prior investigation. It was hypothesized that high doses of the cocktail that did not show performance improvement might be caused by the excessive concentration of the active components such as total phenol, tannin, and saponin, which may decrease nutrient digestion and absorption [7]. After the starter period, the impact of AGPs and PCs in the diet was diminished.

This study aimed to evaluate the impact of a novel blend of natural plant extracts-liquid coconut shell smoke, clove leaf extract, and mangosteen rind extract-on performance, intestinal microbes, and carcass traits of slow growth chickens under various feeding schedules, as a non-antibiotic alternative to AGPs.

## Materials and Methods

### Ethical approval

The animal care and protocols used in this experiment were approved by the Institutional Animal Ethics Committee of ACIAR-IRIAP (Approval number: Balitbangtan/Balitnak/Rm/04/2021.

### Study period and location

This study was conducted from June 2021 to September 2021 at the Poultry Experimental Unit, Indonesian Research Institute for Animal Production, Ciawi Bogor - Indonesia.

### Preparation of the PC

The cocktail consisted of coconut shell liquid smoke, clove leaf extract, and mangosteen peel extract. In Cinangneng Bogor, a home industry produced coconut shell liquid smoke. Clove leaves were sourced from Ciawi-Bogor, West Java, and mangosteen peel from Central Java, both in Indonesia. Clove leaf extract and mangosteen peel were processed using the methods outlined by Pasaribu *et al*. [8]. In brief, clove leaves and mangosteen peel were cut into small pieces, dried in an oven at 40°C–60°C for 4–5 days, crushed, and ground using a laboratory blender. The powder was sieved with size number 50 US mesh (300 microns). 160 g of mangosteen peel or clove leaf powder was mixed with 1400 mL of 96% methanol, shaken for 4 h, allowed to stand at room temperature (28^o^C) overnight, and centrifuged at 11200 × *g* for 10 min at 4°C. The solution was passed through a filter paper and concentrated using a rotary evaporator at 40°C. A 1:1:1 ratio mixture of liquid coconut shell smoke, mangosteen peel extract, and clove leaf extract was used to create the PC.

### Experimental birds and their housing

A total of 240 one-day-old KUB chicks were maintained with continuous lighting in 60 × 120 × 40 cm (11 birds/m^2^) wire cages. The treatments included five groups: a control diet (CD), CD with 50 ppm Zinc bacitracin (AGPs), PC1 providing 198 mL/ton feed from weeks 0 to 12, PC2 offering 198 mL/ton feed from weeks 0 to 4, and PC3 supplying 198 mL/ton feed from weeks 0 to 8. Antibiotics Zn-bacitracin and PC were administered by incorporating them into the feed. There were 6 replications (cages) per treatment and 8 chicks per replicate. The cages came with drinkers and suspended feeders. The study provided chicks with unlimited feed and water access. The nutritional program was structured as two distinct phases: starter (for newborns up to 4 weeks) and grower-finisher (for those over 4 weeks but not yet 12 weeks old). The control and grower diets were formulated according to nutritional recommendations of Sinurat *et al*. [7]. The CD’s composition and formula are given in Table-1. The mash form was used for all experimental diets. Chicks were vaccinated against Newcastle disease, Marek’s disease, and Gumboro disease at the hatchery.

**Table-1:** Ingredient and nutrient compositions of the basal diets.

Item	Starter (0–4 weeks old)	Grower and Finisher (5–12 weeks old)
Ingredient, %		
Corn	50.6	51.81
Soybean meal	26.51	17.49
Wheat Polard	10.00	10.00
Palm kernel meal	5.00	11.00
Crude palm oil	2.030	2.92
Meat and bone meal	4.30	5.36
Limestone	0.86	0.70
L-lysine	-	0.05
DL-Methionine	0.19	0.14
Threonine	-	0.02
Salt	0.20	0.20
Vitamin mineral premixes	0.17	0.17
Toxin binder	0.04	0.04
Total	100	100
Nutrient composition, %		
Dry matter	88.35	89.21
Crude protein	22.25	20.31
Crude fat	6.60	6.33
Gross energy (kcal/kg)	4,103	4,102
Calculated apparent metabolizable energy (kcal/kg)	2900	2800
Crude fiber	5.06	4.92
Ash	5.76	5.44
Ca	1.24	1.26
P_total_	0.61	0.61

One kilogram of calvimix included the following: Vitamin A, 50,000,000 IU; Vitamin D3, 9,000,000 IU; vitamin E, 80,400 mg; vitamin K3, 10,000 mg; Vitamin B1, 10,000 mg; Vitamin B2, 20,000 mg; Vitamin B6, 12,000 mg; Vitamin B12, 100 mg; Vitamin C, 10,000 mg; Ca-d pantothenate 40,000 mg; nicotinamide 120,000 mg; folic acid 4000 mg; biotin, 100 mg; no-carrier

### Parameters measured

Body weight (BW) and feed intake (FI) were recorded weekly while mortality was recorded everyday. The feed conversion ratio (FCR) was determined by dividing the feed consumption by the animal’s weight. Two birds, chosen at random from each pen, were humanely euthanized. Intestinal microbe populations were calculated from intestinal content samples taken from eviscerated birds, and then, the birds were de-feathered. The eviscerated birds, along with their abdominal fat, proventriculus, gizzard, pancreas, spleen, bursa Fabricius, and liver, were all weighed.

### Intestinal total microbial count

Intestinal microbial population was determined by freezing and storing intestinal digesta samples in container tubes. 1 g of intestinal digesta was homogeneously shaken with 9 mL of a sterile distilled water diluent in a test tube to measure the microbial population [9] with slight modifications. Briefly, one gram of intestinal digesta was put into a test tube containing 9 mL of a diluent sterile distilled water and shaken homogeneously, then a new sample (dilution 1) was obtained, which has a population of a microorganism per ml (10^-1^). Dilution 1 was taken 1 ml and added to a second tube containing 9 ml of the diluent, shaken until homogeneous, and a dilution of two (10^-2^) of the original sample was obtained. The dilution was repeated until it reached 10^-10^. Then, 0.1 mL of a 10^−10^ dilution sample was spread on a nutrient agar plate with a sterile glass rod. Each sample underwent the identical procedure thrice. The number of microbial colonies was determined by counting them after an overnight incubation of the plate. To obtain the most accurate estimation of the microbial population, each sample was diluted for a final plate to have between 30 and 300 colonies.

Colony-forming units per gram of sample (log10) were determined by counting bacterial colonies on each plate using a colony counter.

The microbial population was determined using the following formula:

Number of CFUs per ml or per gram of sample


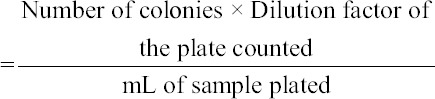


### Statistical analysis

The data obtained in this study were analyzed using one-way analysis of variance (ANOVA) using the General Linear Model procedure of the SAS statistical program (2000, SAS Institute Inc., Cary, NC, USA). Different treatment means were determined using Duncan’s test if the ANOVA was significant at p < 0.05.

## Results

### Growth performance

The growth performance of KUB chickens in response to dietary PC or AGPs supplementation is tabulated in Table-2. In all treatments, the initial BWs of birds were not significantly distinct (p > 0.05). 4-week-old KUB chickens in the AGPs group weighed more significantly (p < 0.05) than those fed PC1 and PC2, while their weight was similar to those fed CD and PC3; PC3 significantly (p < 0.05) outweighed the chickens fed CD. In the AGPs group, FI was similar to that in the PC group (p > 0.05), but different from that in the CD group (p < 0.05). The FCR reveals no significant difference between the AGPs group and PC1 or PC3, but a significant difference with PC2.

**Table-2:** Effect of PC on growth performance of KUB chickens.

Item	DOC BW (g/bird)	Starter (0–4 weeks old)			Grower (0–8 weeks old)			Finisher (0–12 weeks old)		
		
		BW (g/bird)	FI (g/bird)	FCR (g/g)	BW (g/bird)	FI (g/bird)	FCR (g/g)	BW (g/bird)	FI (g/bird)	FCR (g/g)
Treatment										
Control (CD)	27.4 ± 0.21	217.5^bc^ ± 8.22	485.0^a^ ± 1.93	2.233^ab^ ± 0.08	659.8 ± 38.51	1648.6 ± 16.99	2.507^ab^ ± 0.13	1107.6^ab^ ± 58.33	3256.6 ± 78.07	2.945^bc^ ± 0.09
AGPs	27.4 ± 0.37	225.92^ab^ ± 3.79	475.4^ab^ ± 12.50	2.105^bc^ ± 0.05	665.1 ± 21.11	1619.7 ± 38.43	2.436^b^ ± 0.03	1135.7^a^ ± 21.80	3264.2 ± 60.43	2.875^bc^ ± 0.03
PC1	27.5 ± 0.28	212.5^c^ ± 11.18	468.2^b^ ± 11.01	2.208^ab^ ± 0.09	659.5 ± 20.28	1643.9 ± 14.59	2.499^ab^ ± 0.08	1103.5^ab^ ± 32.64	3278.1 ± 55.74	2.972^ab^ ± 0.07
PC2	27.3 ± 0.39	209.7^c^ ± 8.56	474.7^ab^ ± 9.71	2.264^a^ ± 0.11	626 ± 28.78	1630.6 ± 13.23	2.610^a^ ± 0.11	1066.9^b^ ± 44.60	3253.2 ± 84.24	3.053^a^ ± 0.09
PC3	27.2 ± 0.36	231.4^a^ ± 13.47	474.9^ab^ ± 9.58	2.059^c^ ± 0.12	680.5 ± 38.51	1648.2 ± 27.56	2.428^b^ ± 0.11	1158.8^a^ ± 47.78	3293.0 ± 27.98	2.846^c^ ± 0.11
p-value	0.33	0.03	0.02	0.04	0.15	0.72	0.09	0.0447	0.19	0.01

PC=Phytogenic cocktail, AGPs=Antibiotic growth promoters, DOC=Day old chick, BW=Body weight, FI=Feed intake, FCR=Feed conversion ratio, CD=Control diet, AGPs=CD ± Zn-bacitracin, PC1 (CD ± PC 198 mL/100 kg given from starter to finisher [0–12 weeks]), PC2 (CD ± PC 198 mL/100 kg given only as a starter [0–4 weeks), PC3 (CD ± PC 198 mL/100 kg only given from starter-growers [0–8 weeks])

At 8 weeks age, birds that fed PC during the grower period did not significantly differ (p > 0.05) in BW from those fed with AGPs. No significant difference (p > 0.05) in FI existed during the grower phase. At 12 weeks, KUB birds in the PC3 group had comparable BW to that of AGPs (p > 0.05) yet were heavier compared to those in the PC1 and PC2 groups (p < 0.05). Although there was no significant difference (p > 0.05) among all treatments regarding FI in the finisher phase, the FCR in the PC3 group remained on par (p > 0.05) with that in the antibiotic group (AGPs), while PC3 still outperformed PC1 and PC2.

### Effect of PC on intestinal microbes

**Figure-1:**
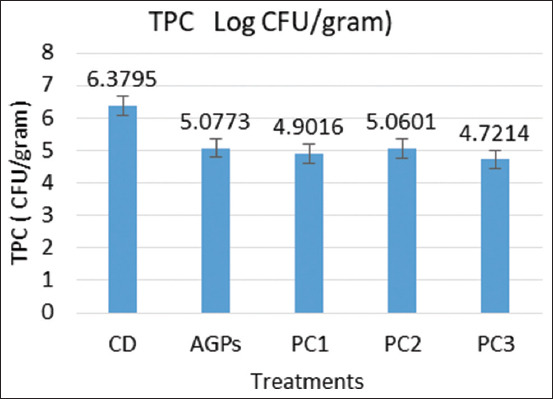
Effect of PC on gut microbes of KUB chickens. CD=Control diet, AGPs=Antibiotic growth promoters, PC=Phytogenic cocktail, AGPs (CD ± Zn-bacitracin), PC1 (CD ± PC 198 mL/100 kg given from 0 to 12 weeks old), PC2 (CD ± PC 198 mL/100 kg given from 0 to 4 weeks old), PC3 (CD ± PC 198 mL/100 kg given from 0 to 8 weeks old).

Figure-1 depicts the intestinal microbiota population of 12-week-old KUB chickens. In the intestinal microbiota population, no significant distinctions (p > 0.05) existed among all treatments. The microbe populations in the PC treatment groups (PC1, PC2, and PC3) did not differ from those in the antibiotic group (AGPs), but were lower than in the control (CD) group.

### Effect of PC on carcass and visceral organ relative weights

The carcass weights of the birds did not significantly differ between the treatments (p > 0.05). The relative carcass weight of the birds showed no significant (p > 0.05) differences between the treatments (Table-3). The treatments significantly affected the relative weight of abdominal fat in the chickens (p < 0.01). Supplementation of AGPs and the PC1 for 0–12 weeks and P C2 for 0–4 weeks significantly (p < 0.01) reduced the abdominal fat level of the chickens. No significant difference was found in the relative weights of organs related to the digestive and immune systems across all treatments (p > 0.05), including the heart, proventriculus, gizzard, pancreas, cecum, spleen, bursa of Fabricius, and liver (Table-3).

**Table-3:** Effect of PC on carcass yield and visceral organs of KUB chickens at 12 weeks of age.

Parameters	CD	AGPs	PC1	PC2	PC3	p-value
Carcass, g/100 g BW	82.43	84.56	78.22	80.78	85.10	0.123
Abdominal fat, g/100 g BW	0.73^d^	1.03^b^	1.15^a^	0.83^c^	0.62^e^	0.001
Organs related to the digestive tract, g						
Crop	8.12	11.35	12.8	9.55	6.93	0.8168
Proventiculus	4.97	5.06	50.52	4.9	5.7	0.7751
Gizzard	30.67	24.6	24.88	25.53	29.66	0.1749
Pancreas	1.93	2.08	2.27	2.05	2.45	0.4474
Cecum	11.65	12.63	13.05	13.18	12.98	0.8413
Organs related to the immune system, g						
Spleen	3.38	2.43	2.33	2.47	2.6	0.5181
Bursa Fabricius	2.02	2.47	2.23	2.5	2.15	0.8564
Heart	6.4	6.55	5.47	5.68	5.93	0.3258
Liver	27.22	23.88	22.9	23.13	25.4	0.3258

AGPs=Antibiotic growth promoters, PC=Phytogenic cocktail, CD=Control diet, AGPs=CD ± Zn-bacitracin, PC1 (CD ± PC 198 mL/100 kg given from starter to finisher [0–12 weeks]), PC2 (CD ± PC 198 mL/100 kg given only as starter [0–4 weeks]), PC3 (CD ± PC 198 mL/100 kg only given from starter-growers [0–8 weeks])

## Discussion

Chickens fed PC from 0 to 8 weeks (PC3) performed best, with results similar to those fed AGPs and continuously fed PC from 0 to 12 weeks (PC1). Supplementing with AGPs or PC during the starter period led to an enhancement in FCR. The FCR enhancement was attributable to a considerable rise in BW, with minimal impact on feed consumption. Performance at 8 and 12 weeks was significantly impaired in the PC2 group, which received no further PC feeding after 4 weeks. However, feeding chickens the PC from 0 to 8 weeks only (PC3) showed the best performance with similar results to those fed the AGPs and those fed the PC continuously from 0 to 12 weeks (PC1). Giving PC to chickens for the first 8 weeks reduces the need for feed additives. At 12 weeks, the PC3 group had a 4.7% greater BW increase than the control, whereas the AGPs group saw only a 2.6% increase. PC inclusion exhibited a comparable impact on FCR. At 12 weeks, the PC3 group had the highest FCR comparable to the AGPs group. The PC3 treatment yielded a 3.36% greater improvement in FCR than CD, and the AGPs treatment yielded a 2.38% greater improvement than the control. From birth to 8 weeks, PC should be supplied to KUB chickens at a rate of 198 mL/100 kg of BW. The information on plant extract supplements given at different ages and feeding regimens is insufficient. The performance of chickens in response to phytogenic feed additives varied. Some reports have shown a positive effect on BW gain (BWG) and FCR, whereas others have shown an increase in BWG without affecting FCR or an increase in FCR due to reduced FI [10]. The PC consists of coconut shell liquid smoke, mangosteen peel extract, and clove leaf extract serving as potent antimicrobial agents [8]. Plants rich in phenol, eugenol, and α-mangosteen can replace antibiotics in poultry feed. Studies suggest that eugenol, mangostin, and phenol enhance broiler chicken performance [11–13]. Broiler chickens aged 1–21 days, those supplemented with 0.50% herbal blend (60% *Anacardium occidentale*, 20% *Psidium guajava*, and 20% *Morinda citrifolia*), showed improvement in FCR in week 2 with no change in BW [14]. Broiler chickens 21–35 day-old grew faster when administered a lower level of *Undaria* extract compared to the stunted growth observed in chickens given a high level of *Undaria* extract [15]. 0.2 mL/L and 0.3 mL/L of herbal extract had varying influences on weight gain among broilers aged 14, 28, and 42 days [16]. Mahfuz *et al*. [17] reported that tannin, polyphenol, saponin, flavonoid, and essential oil from plants stimulate poultry growth at low concentrations. The extract from coconut liquid smoke, clove leaf, and *Garcinia mangostana* pericarp, enriched in phenols, eugenol, and mangostin, respectively, boosted the weight and improved intestinal health of Pekin ducks, as did grape seed extract [18]. The high level of *Undaria* extract administration to chickens caused stunted growth compared with controls, which developed better in the 21–35 day period in broiler chickens [15]. Providing different concentrations of plant extracts to broiler chickens during specific age phases changes their BWGs. Sigolo *et al*. [19] found that chickens fed coriander, dill, or thyme extracts, at 150 mg/L from ages 0 to 14, had greater BWGs than those given the higher 450 mg/L dosage. The mixture of carvacrol, capsaicin, and cinnamaldehyde plant extracts at 75, 150, and 225 ppm did not impact broiler BW [20]. According to Kumar *et al*. [21], chicken performance and FCR were enhanced by plant extract supplements containing 100 ppm eugenol and garlic tincture in chicken rations. 2% dose of mangosteen peel extract did not impact BW, FI, or FCR in broiler chickens [11]. 1.5% liquid smoke encapsulation in the feed enhances weight gain and feed conversion rate, but not FI, in livestock [22].

The development of microbes in the digestive tract is influenced by the availability of nutrients. Only certain microbes in the digestive tract are affected by bioactive substances. Abdelli *et al*. [10] concluded that phytogenics influence the microbial community in broiler digestive tracts. The effect of phytogenics on chickens’ gut microbiota can vary from neutral to beneficial, contingent on their composition, and inclusion levels. All treatments did not yield statistically significant differences in this experiment (p > 0.05). The extract of marjoram plant decreased the overall bacterial population in chickens’ intestines [23]. PC3 treatment, administered at 198 mL/100 kg of feed for the first 8 weeks of life, reduced intestinal microbe population by 26%. Among all treatments, the same one yielded the top results for both BW and FCR. The decrease in intestinal microbes may contribute to improved chicken performance. The antibacterial properties of PC (coconut shell liquid smoke, mangosteen peel extract, and clove leaf extract) have been demonstrated through *in vitro* studies to suppress the growth of pathogenic *E. coli* [8]. Marjoram plant extract also reduced the total bacterial count in the intestines of chickens [23]. Pathogenic intestinal bacteria can be suppressed by clove essential oil [4].

The relative weight of KUB chicken carcasses was not significantly influenced by treatment. Some studies have shown that supplementation of feed with a blend of plant extracts consisting of turmeric, citrus, and grape seed extract, Chinese cinnamon essential oil, chile boldo leaves, fenugreek seeds, and organic acids blended in the feed also increased the carcass of broiler chickens [24]. A study revealed no significant improvement in carcass yield for broilers fed vegetable extract (oregano, cinnamon, and cloves) at concentrations of 100 g/ton and 150 g/ton) [25].

During the first 12 weeks of life, PC was fed continuously (PC1), which led to a 58% increase in abdominal fat compared to control (CD). The KUB chickens in this study had an abdominal fat level of 10.0–12.4 g/kg BW, comparable to the reported range of Sinurat *et al*. [26]. The PC3 group had the smallest abdominal fat relative weight (15% lower than the CD group, 39% lower than AGPs group). Feeding PC during the whole growing period from 0 to 12 weeks (treatment PC1) also increased abdominal fat 58% higher than CD. Supplementation with AGPs raises abdominal fat levels by about 40%. AGPs activation in the liver initiates abdominal fat deposition by triggering fat synthesis [27]. Use of PC instead of AGPs as feed additives from 0 to 8 weeks in chickens result in decrease in abdominal fat. Mangosteen peel extract supplementation in the diets of slow growth chickens does not influence abdominal fat levels [28]. The impact of phytogenic supplements on abdominal fat deposition in chickens depends on feeding regimes. The research revealed that feeding phytogenics decreased abdominal fat from weeks 0 to 8 but increased it from weeks 0 to 12. Several studies failed to detect an impact on chickens’ liver, spleen, and thymus weight when they were fed a plant extract containing bioactive compounds [29–31]. Reports indicate an increase in bursa of Fabricius weight when grape pomace concentrate is included in broiler chicken feed [31]. The immune system’s visceral organ’s weight remained unchanged by the treatment. Several studies found no effect of providing a plant bioactive substance mixture on the weight of the liver, spleen, and thymus of chicken [31, 32, 33]. However, Zhang *et al*. [31] reported an increase in bursa of Fabricius weight as an effect of plant bioactive inclusion.

## Conclusion

PCs added to KUB chicken feed enhance their performance. The optimal feeding regimes yielded the best outcomes between the ages of 0 and 8 weeks. In the 0–4 week feeding period, PC performance deteriorated, while no improvement was seen in the 0–12 week period. The application led to an increase in BW and FCR while reducing abdominal fat. Based on the study results, PC can replace AGPs as feed additives, since it showed similar or better improvement.

## Authors’ contributions

TP and APS: Designed the study, collected and interpreted the data, and drafted the manuscript. MS, and JAL: Analysis of the data and drafted the manuscript. All authors have read, reviewed, and approved the final manuscript.
